# Growth discordance of monoamniotic twin because of difference of cords diameter in forked umbilical cord: Case report

**DOI:** 10.1097/MD.0000000000008042

**Published:** 2017-09-15

**Authors:** Yongbing Guo, Yu Sun, Huixia Yang

**Affiliations:** Obstetrics and Gynecology Department, Peking University First Hospital, Beijing, China.

**Keywords:** cord diameter, forked umbilical cord, monoamniotic twin, twin growth discordance

## Abstract

A case of monochorionic-monoamniotic (MCMA) twin pregnancy with growth discordance because of difference of cord diameter in forked umbilical cord is reported.

MCMA twins were diagnosed at 12 weeks of gestation and twin growth discordance was considered during the follow-up twice-weekly visits to the ultrasound and prenatal care units. The pregnancy was terminated at 34 weeks. Two live female babies weighing 2510 g and 1940 g were delivered. Examination of placenta and umbilical cords after birth showed that the 2 cords merged into a conjoint cord 1 cm from insertion to the placenta (forked umbilical cord). Placental color injection showed that the 2 fetuses shared the same placenta area. The diameters of the 2 cords were significantly different (1.5 vs 0.8 cm). This caused an unequal distribution of blood and nutrients, which is the real reason of twin growth discordance in this case.

This case reveals that the diameter discordance of cords can be an important factor for twin growth discordance. Few relevant cases have previously been reported. Cords diameter measurement is suggested for ultrasound surveillance of twin growth discordance.

## 1 Introduction

Monochorionic-monoamniotic (MCMA) twins are the rarest type of twin pregnancy. MCMA is associated with a significantly high perinatal morbidity and mortality rate, which has been reported as 10% to 40%.^[1]^ Birth weight discordance is an independent predictor of perinatal mortality in twin pregnancy.^[2]^ Accurate prenatal diagnosis, intensive fetal surveillance, timed cesarean delivery, and improvement of neonatal care can reduce the perinatal mortality rate.^[3]^ Discordance is measured as (larger estimated or actual weight − smaller estimated or actual weight/larger estimate or actual weight).

Because of the lack of consensus on the precise threshold of discordance that is linked with complications, the American Congress of Obstetricians and Gynecologists considers a 15% to 25% difference in actual weight between twins to be discordant.^[4]^ The consensus statement by the Society of Obstetricians Gynecologists of Canada defined discordance as a difference of abdominal circumference (AC) of 20 mm or estimated fetal weight (EFW) difference of 20%.^[5]^

The etiology of growth discordance in twins has been extensively investigated, and it is related to the chorion. In monochorionic twins, differences are largely attributed to the vascular anastomosis, inequalities in distribution of placental mass between the 2 fetuses and abnormalities in cord insertion site.^[6]^ Cord abnormalities include abnormal insertion and abnormal growth, such as marginal, velamentous cord insertions, and a single umbilical artery (UA). Cord diameter problems have seldom been considered.

In this article, we describe the case of a 26-year-old Chinese woman diagnosed with MCMA twins at 12 weeks of gestation. She developed twin growth discordance in the second trimester. Placental injection revealed that the 2 cords were conjoined into a single cord 1 cm from the insertion into the placenta. The diameters of the 2 cords were significant different, which produced the growth discordance. This conclusion has not been reported before.

## 2 Case report

This study was approved by the ethic committee of Peking University First Hospital. Informed consent was obtained from all individual participants included in the study. The patient was 26-year-old Chinese woman, gravid 1 para 0, with an unremarkable past medical, surgical and family history. She was referred to our hospital for investigations and management when she found out she was pregnant.

At 12 weeks of gestation, we took a detailed ultrasound examination that confirmed intrauterine MCMA twin gestation with the twin umbilical cord entanglement. The umbilical cords of the 2 fetuses were very close to each other in the insertion into the placenta, almost side-by-side. Crown-lump length (CRL) of the fetus A and B was 60 mm and 55.7 mm, respectively, which represented a difference of 7.2%. To strengthen the management of the monoamniotic twins, follow-up examinations were recommended, with ultrasound examinations every 2 weeks. From 17 weeks of gestation, the AC difference of 2 fetuses became increasingly significant based on the ultrasound data, ranging from 18.6 to 42.6 mm. We calculated the EFW of the 2 fetuses beginning at 27 weeks of gestation. The EFW difference was maintained at 24% to 25%. We considered the complication of twin growth discordance. Therefore, we paid more attention to the UA and middle cerebral artery peak systolic velocity (MCA PSV) color Doppler of the 2 fetuses. The UA and MCA PSV were essentially normal during the entire gestation. And the observations of positional relation of umbilical cords were performed regularly, until delivery. Besides we calculated the cords diameters of the twin in the third trimester. At 29 weeks of gestation, the cord diameter of fetus A was17.86 mm, and cord diameter of fetus B was12.78 mm. And at 33weeks of gestation, the diameters of the 2 cords were significantly different (19.62 vs 12.78 mm).

At 33 weeks of gestation, an ultrasound examination revealed an EFW of fetus A and B of 2300 g and 1750 g, respectively. The difference was 24%. In consideration of MCMA twin, pregnancy should be terminated at 32 to 34 weeks according to the newest ACOG PRACTICE BULLETIN about multiple gestations and the Guideline for twin pregnancies of China (part 1): antenatal care and management for uncomplicated twins.^[7,8]^ We suggested that the patient take dexamethasone to promote fetal lung maturation before parturition. Five milligrams of dexamethasone was administered 4 times in 48 hours for fetal lung maturity acceleration as the preemptive attempt. A caesarian delivery was done at 34 weeks.

Two live female babies weighing 2510 g and 1940 g were delivered. The Apgar score in both was 10. The difference of the actual weight between the 2 babies was 22.7%, which confirmed twin growth discordance. The babies were transferred to the neonatal intensive care unit (NICU) because of their premature birth. The examination of the placenta and umbilical cords showed that the 2 cords merged into a conjoint cord 1 cm from the point of insertion into the placenta. The 2 cords were entangled but no true knot was found. The diameters of the 2 fetuses were significantly different; 1.5 cm for larger birth weight baby and 0.8 cm for the smaller birth weight baby.

**Figure 1:**
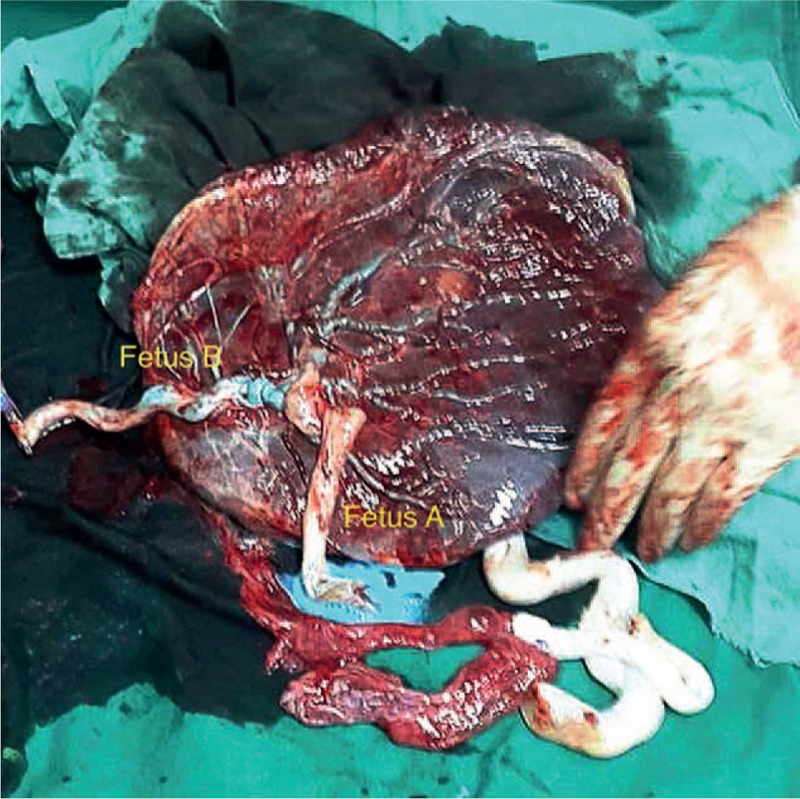
Blue stained surface veins in the placenta after placental injection.

**Figure 2:**
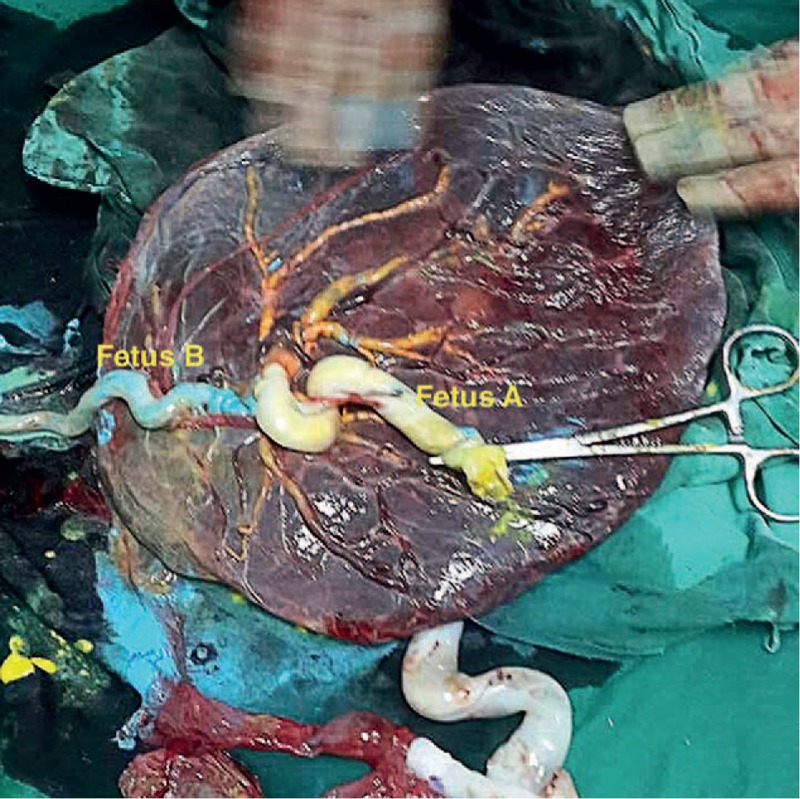
Yellow stained surface veins in the placenta of after placental injection.

**Figure 3:**
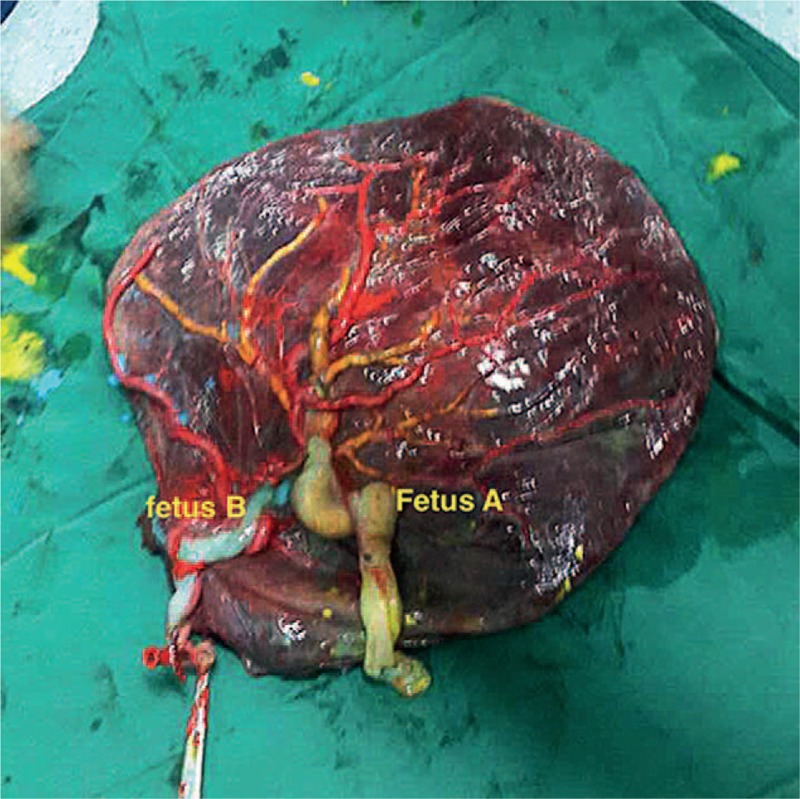
Red stained surface arteries in the placenta after placental injection.

The conjoint cord was actually a single forked cord. When blue dye was injected in the umbilical vein of fetus B the surface vein in the placenta became blue (Fig. 1). When yellow dye was injected into the umbilical vein of fetus A, the blue vessel immediately became yellow (Fig. 2). Finally, when red dye was injected into one UA of fetus B, the surface artery becomes red (Fig. 3). We failed to inject color dye into the UA of fetus A because of considerable arterial resistance.

## 3 Discussion

A forked umbilical cord is a rare and unique phenomenon in monoamnioatic twin gestations. Fraser et al^[9]^ reported the first case of live born monoamniotic twins with a bifurcated umbilical cord 20 years ago. Frishch et al^[10]^ also reported a case about forked umbilical cord, but micro-observation showed that the forked umbilical cord had 2 independent umbilical veins and 4 UAs. Therefore, in that case there were actually 2 independent cords, but they apparently merged into 1 when observed by the naked eye.

In our case, because of the extremely rare possibility that the 2 cords of the twins merged into a single cord close to the insertion of placenta was not considered, we failed to measure the actual distance between roots of the 2 umbilical cords in the first trimester. We found the 2 cords were very close to each other with cord entanglement. We eventually found the 2 umbilical cords conjoined into one umbilical cord 1 cm from the placental insertion site by examination of the placenta and confirmed that it was one single forked umbilical cord by placenta injection. So, in fact the twins shared one common placenta and had a uniform blood circulation at the same time. Thus, the case did not feature the aforementioned situations of sharing of different placenta, anastomosis of the placenta and an abnormality of the umbilical cord. This is obviously different from previous reports about the pathogeny of twin growth discordance. Through measurements of the placenta and umbilical cord, we found a significant difference between the umbilical cord diameters of the 2 babies (1.5 vs 0.8 cm).

We conclude that in this case the discordance in growth of the 2 fetuses was caused by the significant difference in the umbilical cord diameters. There was no difference in sharing of the placenta or anastomoses of placenta between the 2 babies. However, the diameter of the umbilical cord of the larger birth weight baby was almost twice as big compared with the smaller birth weight baby. Thus, before birth the larger fetus would have received more blood and nutrients, which were beneficial for growth. Conversely, the growth and development of the other fetus would have been restricted by the relatively diminished nutrition. The resulting growth discordance has been rarely reported.

## 4 Conclusion

The diameter discordance of umbilical cords of twins is an important factor for twin growth discordance. This highlights the need for increased attention to the distance between the insertion sites of twin fetuses and the need to focus on the diameters of the umbilical cords in ultrasonic testing in cases of MCMA. These steps will help detect umbilical cord abnormalities and aid in the prediction of the possibility of growth discordance of the twins. The clinical outcome might also be beneficially affected. Earlier detection of problems would aid individual antenatal care management.

## Acknowledgments

The authors would like to acknowledge the financial support from the Beijing Key Laboratory of Maternal Fetal Medicine (no. Z151100001615051) and the 973 National Science and Technology Plan Project of China (no. 2015CB943304).
